# Electrocardiographic Features in Transthyretin Cardiac Amyloidosis

**DOI:** 10.3390/diagnostics16010065

**Published:** 2025-12-24

**Authors:** Shunsuke Kiuchi, Shinji Hisatake, Hidenobu Hashimoto, Yoshiki Murakami, Takanori Ikeda

**Affiliations:** Division of Cardiovascular Medicine, Department of Internal Medicine, Toho University Faculty of Medicine, Tokyo 143-8541, Japan; s-hisatake@med.toho-u.ac.jp (S.H.); hidenobu.hashimoto@med.toho-u.ac.jp (H.H.); yoshiki.murakami@med.toho-u.ac.jp (Y.M.); takanori.ikeda@med.toho-u.ac.jp (T.I.)

**Keywords:** transthyretin cardiac amyloidosis, ^99m^Tc-labeled pyrophosphate scintigraphy, left ventricular hypertrophy, electrocardiogram

## Abstract

**Background:** ^99m^Tc pyrophosphate scintigraphy (^99m^Tc-PYP) is useful for diagnosing transthyretin amyloid cardiomyopathy (ATTR-CA). We examined the characteristics of ^99m^Tc-PYP-positive patients at our institution. **Methods:** A total of 76 patients who underwent ^99m^Tc-PYP from December 2020 and March 2022 were grouped into ^99m^Tc-PYP-positive (P) and -negative (N) groups and compared. **Results:** Nine of seventy-six patients were positive (11.8%), and all patients were diagnosed with ATTR-CA by myocardial biopsy or clinical findings. The heart-to-contralateral lung ratio in the P group was significantly higher (N Group: 1.15, P Group: 1.92, *p* < 0.001). In the P group, the left ventricular posterior wall thickness was significantly thickened (N Group; 12.5 mm, P Group; 15.5 mm, *p* = 0.003). Electrocardiogram showed left ventricular hypertrophy (LVH) was observed more frequently in the N group (N Group; 30 patients (44.8%) and the P Group; 1 patient (11.1%), *p* < 0.001). In addition, the QTc interval was significantly prolonged in the P group (N Group; 422 msec, P Group; 456 msec, *p* = 0.001). **Conclusions:** In patients who have significant LVH on echocardiogram but not on electrocardiogram, ^99m^Tc-PYP may be useful for diagnosing ATTR-CA. However, the present study is a single-center retrospective study with a small number of patients, and the results are exploratory and hypothesis-generating. Prospective studies with a larger number of subjects are needed.

## 1. Introduction

Traditionally, transthyretin amyloid cardiomyopathy (ATTR-CA) was considered a rare disease. In 2018, the efficacy of tafamidis for ATTR-CA was reported [1], and in March 2019, the use of tafamidis for ATTR-CA became covered by insurance in Japan. The usefulness of ^99m^Tc pyrophosphate scintigraphy (^99m^Tc-PYP) in the diagnosis of ATTR-CA has been described in the guidelines of the Japanese Circulation Society [2], and its diagnosis and treatment methods are improving year by year. In particular, ^99m^Tc-PYP is an examination with extremely high sensitivity and specificity. Due to these advances in diagnosis and treatment, the number of ATTR-CA patients in the United States and Japan is increasing year by year [3,4]. Furthermore, ATTR-CA patients have been reported in Tokyo and Hokkaido, outside of Kumamoto and Nagano, where hereditary ATTR-CA is concentrated in Japan, so it may no longer be a rare disease. In ATTR-CA, the transthyretin tetramer is unstable, making it easy for amyloid to accumulate in tissues. Tafamidis binds to and stabilizes the transthyretin tetramer, preventing its dissociation into monomers and inhibiting the formation of amyloid fibrils and their deposition in tissues. The 30-month survival rate of ATTR-CA patients was approximately 60%, however the ATTR-ACT study reported that the use of tafamidis increased the survival rate to approximately 70%, and the prognosis improved after that [5]. On the other hand, tafamidis is a medicine that suppresses the formation of amyloid fibrils and their deposition in tissues, however, it does not reversibly improve the disease, so early introduction is desirable. The ATTR-ACT study also reported that patients with New York Heart Association (NYHA) stage III or IV had a worse prognosis than those with stage I or II [6]. Therefore, early introduction of these medications of ATTR-CA is essential, and early diagnosis of ATTR-CA is important. ^99m^Tc-PYP is useful for diagnosing ATTR-CA, but the number of patients in which it can be performed is limited due to its high cost and the length of time required for the examination. If it were possible to evaluate patients in which ^99m^Tc-PYP should be performed using clinically easy-to-use indicators such as electrocardiograms, this would lead to early diagnosis and would be useful. Therefore, in the present study, we used ^99m^Tc-PYP to compare the characteristics of positive and negative patients.

## 2. Materials and Methods

The present study was a single-center retrospective observational study in accordance with the Declaration of Helsinki (their medical records were accessed for data collection) and was approved by the Ethics Committee of Toho University Omori Medical Center (approval number: M24196 23100, approval date: 25 February 2025). The details about the present study were disclosed in opt-out format on the website of our institution and our department (Department of Cardiovascular Medicine) (granted a waiver of informed consent from study participants). The subjects of the present study were given the opportunity to decline to be enrolled.

### 2.1. Study Participants

Seventy-six consecutive patients (aged 32–89 years) who underwent ^99m^Tc-PYP for suspected cardiac amyloidosis at our institution between December 2020 and March 2022 were enrolled in the present study. ^99m^Tc-PYP was performed primarily on patients basically with a history of hospitalization for heart failure (HF), with increasing left ventricular wall thickness of 12 mm or more or in whom the attending physician suspected cardiac amyloidosis based on clinical findings. In addition to left ventricular wall thickening, if there is HF, abnormal electrocardiographic findings, aging, blood examination or imaging findings, or medical history, a diagnosis of wild-type ATTR-CA using ^99m^Tc-PYP is also recommended. At our institution, the standard for ^99m^Tc-PYP is for attending physicians to identify patients who are candidates for disease-modifying medications, in addition to conditions such as carpal tunnel syndrome [7].

### 2.2. Study Outcomes

The aim of the present study was to compare the patient backgrounds of the ^99m^Tc-PYP-positive group (P Group) and the negative group (N Group) and to investigate factors associated with ^99m^Tc-PYP-positive patients.

### 2.3. Data Collection

We evaluated the patient’s baseline clinical characteristics, medical history, comorbidities, physical findings, laboratory examinations, electrocardiographic findings, transthoracic echocardiographic findings (TTE), and classification of Kumamoto criteria [8], using electronic medical records.

### 2.4. Patient Clinical Profiles

Age, gender, height, weight, body mass index, blood pressures (systolic and diastolic), and pulse rate were evaluated. We investigated the presence or absence of hypertension (HT), diabetes mellitus (DM), and chronic kidney disease (CKD) as medical history and comorbidities. HT, DM, and CKD were assessed based on medication history or respective guidelines. Moreover, we assessed the severity of HF, using degree of symptoms (New York Heart Association (NYHA) classification) and brain natriuretic peptide (BNP).

### 2.5. Physiological Examinations

Twelve-lead electrocardiography and TTE were performed by two physicians and technicians who were blinded to the present study. In electrocardiography, we evaluated the presence or absence of left ventricular hypertrophy (LVH) and low voltage, as well as intraventricular conduction (PQ duration, QRS duration, QTc duration, and the presence of first-degree atrioventricular block and left bundle branch block). LVH was defined as a Sokolow–Lyon voltage criteria (RV5(6) + SV1 ≥ 35 mm) [9]. The durations of PQ, QRS, and QTc were measured automatically. QTc was calculated with the correction formula with the Bazett method [10]. First-degree atrioventricular block was defined as a PQ of 200 msec or more. Cardiac size, wall thickness, and left ventricle systolic function (ejection fraction: EF) were evaluated by TTE. EF was calculated using either the modified Simpson method (apical two- or four-chamber view) or the Teichholz method (parasternal long-axis view) [11]. Wild-type ATTR-CA has been reported to account for 13% of HF with preserved EF (HFpEF) patients with left ventricular wall thickness ≥ 12 mm [12], and HFpEF of ATTR-CA transitions to HFrEF as the disease progresses. Therefore, we also evaluated the proportion of patients with HFpEF, defined as EF > 50% [13]. In addition, wall thickness was also evaluated because wall thickness does not decrease with disease progression.

### 2.6. Statistical Analysis

Data were presented as median or percentage. A Mann–Whitney U test was used to compare between two groups. *p* < 0.05 was considered statistically significant in all analyses. EZR (Saitama Medical Center, Jichi Medical University), which is a graphical user interface for R (version 2.13.0, The R Foundation for Statistical Computing, Vienna, Austria), was used for statistical analyses.

## 3. Results

### 3.1. ATTR-CA Diagnosis and ^99m^Tc-PYP Positivity Rate

Of the 76 patients, 9 patients were ^99m^Tc-PYP-positive (11.8%). Of the nine positive patients, two patients were diagnosed with ATTR-CA by skin biopsy and six patients by myocardial biopsy. Of the six patients with positive myocardial biopsy results, three patients were diagnosed with ATTR-CA by myocardial biopsy, although the diagnosis was negative using a skin biopsy. All patients diagnosed with ATTR-CA by biopsy were found to have wild-type (non-hereditary) ATTR-CA by genetic testing. One patient was scheduled to undergo a biopsy but died of cerebral hemorrhage before the biopsy could be performed. In this patient, M protein was negative and myocardial accumulation was confirmed on the SPECT image of ^99m^Tc-PYP, thus, under the current statement from the Japanese Circulation Society, ATTR-CA could be diagnosed. Furthermore, the heart-to-contralateral ratio (H/CL ratio) for ^99m^Tc-PYP was significantly higher in the P group (N Group: 1.15, P Group: 1.92, *p* < 0.001). Using the Kumamoto criteria (0/1/2/3) to predict the patients with ^99m^Tc-PYP positivity, the ratio was 37/28/2/0 in negative patients and 0/6/3/0 in positive patients, showing a significant difference (*p* < 0.001).

### 3.2. Physical Findings, Comorbidity, and Laboratory Examinations

Table 1 summarizes the patient characteristics. Age was significantly higher in the P group (N Group; 61 years old, P Group; 81 years old, *p* < 0.001). Height was significantly shorter in the P group. On the other hand, body weight and body mass index (BMI) had no differences. There were no differences between the two groups in past medical history, including hypertension, and blood pressures were similar. NYHA, which indicates the severity of HF subjective symptoms, and brain natriuretic peptide (BNP) in blood examination between the two groups showed no difference (Table 1 and Table 2). BNP is also affected by obesity, renal function, and gender [14,15], however, there were no differences in these factors either (Table 2), and the severity of HF is considered to be at the same level.

### 3.3. Clinical Physiological Function Examination Findings

The electrocardiographic findings are summarized in Table 3. There was no significant difference in the prevalence of atrial fibrillation (af) or atrial flutter between the two groups. There was also no difference between the two groups in low voltage, which is characteristic of cardiac amyloidosis. On the other hand, LVH as shown by the Sokolow–Lyon voltage criteria was significantly more prevalent in the N group (N Group; 30 patients (44.8%) and the P Group; 1 patient (11.1%), *p* < 0.001). The rate of first-degree atrioventricular block in the P group was significantly higher than the N group (N Group; 13 patients (19.4%) and the P Group; 3 patients (33.3%), *p* < 0.001). The proportion of left bundle branch block and intraventricular conduction duration (PQ interval, QRS interval), which indicate intraventricular conduction, tended to be impaired in the P group, and QTc interval was significantly prolonged in the P group (N Group; 422 msec, P Group; 456 msec, *p* = 0.001). The mean and standard deviation of the QTc interval were 471 ± 66 msec in the P group and 430 ± 30 msec in the N group.

Table 4 summarizes the findings of TTE. No differences were observed in cardiac chamber dimensions such as left atrial or left ventricular diameter (diastolic or systolic). There was also no difference in left ventricular systolic function, and the proportions of patients with HFpEF were similar. A significant difference was observed in posterior wall thickness at end diastole (N Group; 12.5 mm, P Group; 15.5 mm, *p* = 0.003).

## 4. Discussion

### 4.1. Main Results and ATTR-CA Diagnosis

In the Japanese guidelines, biopsy was mandatory for the diagnosis of ATTR-CA at the time of study implementation [2], however, the current statement also describes conditions under which biopsy is not necessary. In the present study, ^99m^Tc-PYP-positive patients were judged to have ATTR-CA based on clinical findings, and all of them showed accumulation of ^99m^Tc-PYP in the heart on SPECT images as well as planar images. Therefore, the current statement also diagnoses ATTR-CA. In the Japan Cohort Study of Systemic Amyloidosis (J-COSSA) (currently ongoing), which began in October 2019, approximately 4000 patients of ATTR-CA have been reported nationwide.

Compared to the population of Ota-ku, where our hospital is located, the diagnosis of nine patients of ATTR-CA in one year and four months at our hospital may be somewhat low. For this disease, which requires early treatment, selection of patients who require ^99m^Tc-PYP for early diagnosis is extremely important. In the present study, intraventricular conduction disorder (QTc prolongation) on electrocardiograms and more significant increased wall thickness on TTE were observed in the P group, and LVH on electrocardiograms was significantly less in the P group. Furthermore, the scores of the Kumamoto criteria were significantly higher in the P group.

### 4.2. Electrocardiogram Findings in the ATTR-CA Diagnosis

It has been reported that cardiac amyloidosis, including ATTR-CA, may show low voltage, pseudo-infarction pattern, atrioventricular block, and af on electrocardiogram [2]. Low voltage is reported in 13–40% of wild-type ATTR-CA patients, which is not particularly high [2], and no difference in low voltage was observed in the present study. On the other hand, low voltage was observed in two patients in the present study, one of which was ATTR-CA, indicating high specificity. In patients of af, it has been reported that low atrial voltage was observed in intracardiac electrical potentials in patients with ATTR-CA [16]. Although no difference was observed in the present study, it has been reported that af is more common in ATTR-CA [17], and attention should also be paid to low voltage and concomitant af. Regarding electrical conduction within the heart, results in the P group may suggest impairment. Although the Kumamoto criteria use the QRS [8], some previous studies suggest that intracardiac conduction disorders occur in ATTR-CA. In a patient at our hospital for whom it took time to obtain consent for a myocardial biopsy and two years for a definitive diagnosis of ATTR-CA, conduction disorders (widening of QRS waves) progressed over time, and the patient presented with af and complete atrioventricular block [18].

The proportion of patients with LVH on electrocardiograms was notably high in the present study. Both groups had left ventricular wall thickening of 12 mm or more in TTE, however, the P group had a significantly higher incidence of LVH on electrocardiograms. As the heart adapts to pressure overload, myocardial remodeling occurs, and LVH appears on electrocardiogram. LVH on electrocardiogram is associated with the prognosis of hypertensive patients [19]. On the other hand, it has also been reported that electrocardiogram is not suitable for excluding hypertensive cardiac hypertrophy [20]. In patients where significant LVH on electrocardiogram is present, such as in the subjects of the present study, it is assumed that, if they have hypertensive heart disease, they have been exposed to hypertension for a long period of time. It is thought that a higher proportion of patients in the N group had structural cardiac abnormalities due to hypertension and that this resulted in a higher proportion of patients with LVH on electrocardiograms. On the other hand, in ATTR-CA, TTR tetramers become unstable and dissociate to form monomers, forming amyloid fibrils, and the accumulation of amyloid protein in the interstitial space of the heart causes left ventricular wall thickening. The difference in the frequency of increased wall thickness due to amyloid infiltration on electrocardiograms from the present study is thought to be due to the pathology of left ventricular wall thickening.

### 4.3. ATTR-CA and Left Ventricular Hypertrophy

LVH was significantly more prevalent in the N group on electrocardiograms, however, left ventricular wall thickness in the P group tended to be thicker on echocardiograms. In our hospital, ^99m^Tc-PYP was performed when left ventricular wall thickness was 12 mm or more, and in the P group it was approximately 16 mm. In particular, the posterior wall thickness was significantly thickened, and the Kumamoto criteria also use the posterior wall thickness. Additionally, the rate of HFpEF and the degree of EF were similar in both groups. In conclusion, patients in which echocardiography reveals significant increased left ventricular wall thickness but electrocardiograms do not reveal LVH may indicate ATTR-CA, and ^99m^Tc-PYP may be beneficial. However, the present study was a retrospective study based on medical records, therefore it has limitations. One of these is left ventricular strain in echocardiographic examinations. It has been reported that global longitudinal strain (GLS) was useful for detecting ATTR-CA that could not be detected by the Kumamoto criteria [8]. In general, the usefulness of left ventricular strain has been reported [21], and left atrial strain analysis is also known to be effective [22]. However, the present study was a retrospective study and these strain analyses could not be performed.

The present study demonstrated that the absence of LVH on electrocardiograms and a mismatch in wall thickening on echocardiography may be related to the diagnosis of ATTR-CA. Previously, the concept of a low QRS voltage-to-LV mass or wall thickness ratio based on electrocardiogram and echocardiographic findings has also been proposed [23]. This manuscript also demonstrated an inverse correlation between QRS voltage and myocardial mass in patients with amyloidosis. This concept was also useful in patients with amyloidosis accompanied by bundle branch block [24]. The AC-TIVE study, which is a multicenter prospective study in Italy, also reported that the combination of low QRS voltage and a left ventricular septal wall thickness of 16 mm or more had a specificity and positive predictive value of 100% [25]. However, only a small number of subjects in the present study had low QRS voltage, and the number of cardiac amyloidosis patients exhibiting low QRS voltage is not necessarily large. In such patients, it may be useful to focus on the absence of LVH on electrocardiogram due to pressure overload, as in the results of the present study.

### 4.4. Sudden Cardiac Death in ATTR-CA

ATTR-CA causes arrhythmias such as af and conduction disorders, as in the patient of our hospital mentioned above, and is known to be one of the factors in sudden cardiac death (SCD) due to ventricular arrhythmias [26]. The use of an implantable cardioverter defibrillator (ICD) for cardiac amyloidosis has been established as a secondary prevention method, but not as a primary prevention method [27]. SCD is more common in AL-type cardiac amyloidosis than in ATTR-CA [27], however, ICD is more useful in ATTR-CA with hereditary and left ventricular systolic dysfunction [28]. Since SCD is generally more common in hereditary diseases [29], it is important to evaluate the hereditary nature of ATTR-CA when it is diagnosed, and it is essential to evaluate the hereditary nature when applying for an intractable disease in Japan. On the other hand, wild-type ATTR-CA is now commonly detected due to advances in diagnostic methods and treatment in the wild type. There are still many unknowns, such as the use of ICD for the primary prevention of SCD, for which further clarification is awaited.

### 4.5. Study Limitations

The biggest limitation of the present study is the small number of subjects, as it was a retrospective study at a single institution. The P group included only nine patients, an insufficient number of patients to perform multivariate analysis. MRI and CT scans are also useful for diagnosing ATTR-CA [30,31], however, the present study was retrospective, and the number of patients was insufficient. GLS, which is considered useful, also could not performed in this retrospective study. Furthermore, biopsies including myocardial biopsies were not performed in all patients. It is also possible that the data were insufficient to diagnose infiltrative cardiomyopathy, including diagnosis of amyloidosis in PYP-negative patients, in the study populations. To resolve these problems, it is necessary to conduct prospective studies on a large number of patients in which examinations such as MRI, GLS, and myocardial biopsy have been performed.

## 5. Conclusions

In patients who have significant LVH on echocardiogram but not on electrocardiogram, ^99m^Tc-PYP may be useful for diagnosing ATTR-CA. However, the present study is a single-center retrospective study with a small number of patients, and the results are exploratory and hypothesis-generating. Prospective studies with a larger number of subjects are needed.

## Figures and Tables

**Table 1:** Patient backgrounds between groups.

	^99m^Tc-PYP-Negative Group N Group (*n* = 67)	^99m^Tc-PYP-Positive Group P Group (*n* = 9)	*p*-Value
Age (years)	61	81	<0.001
Male (*n*, %)	52, 77.7	6, 66.6	0.475
Height (cm)	164	155	0.009
Weight (kg)	66.4	56.2	0.151
Body mass index (kg/m^2^)	24.4	23.3	0.599
NYHA class (I/II/III/IV)	2/56/9/0	0/9/0/0	0.432
Hypertension (*n*, %)	58, 86.6	8, 88.8	0.849
Diabetes mellitus (*n*, %)	20 29.9	1, 11.1	0.243
Dyslipidemia (*n*. %)	19, 28.4	4, 44.4	0.331
Chronic kidney disease (*n* %)	26, 38.8	3, 33.3	0.755
Systolic blood pressure (mmHg)	122	132	0.186
Diastolic blood pressure (mmHg)	72	78	0.268
Pulse rate (bpm)	79	73	0.290

NYHA: New York Heart Association. Continuous data are expressed as the median. *p*-values were determined using the Mann–Whitney U test.

**Table 2:** Laboratory findings between the two groups.

	^99m^Tc-PYP-Negative Group N Group (*n* = 67)	^99m^Tc-PYP-Positive Group P Group (*n* = 9)	*p*-Value
Sodium (mg/dL)	138.5	138.1	0.751
Potassium (mg/dL)	4.2	4.1	0.570
C-reactive protein (mg/dL)	0.22	0.39	0.650
Total protein (mg/dL)	7.1	7.1	0.991
Total bilirubin (mg/dL)	0.6	0.8	0.144
Aspartate aminotransferase (IU/L)	24.7	29.9	0.339
Alanine aminotransferase (IU/L)	22.4	22.2	0.981
Lactate dehydrogenase (IU/L)	246	274	0.254
Blood urea nitrogen (mg/dL)	20.5	26.6	0.233
Creatinine (mg/dL)	1.20	1.14	0.746
eGFR (mmL/min/1.73 m^2^)	50.1	58.0	0.394
White blood cell (/µL)	6300	6700	0.598
Hemoglobin (g/dL)	12.7	13.2	0.501
Brain natriuretic peptide (mg/dL)	672	514	0.545

eGFR: estimated Glomerular Filtration Rate. Continuous data are expressed as the median. *p*-values were determined using the Mann–Whitney U test.

**Table 3:** 12-lead electrocardiographic examinations between the two groups.

	^99m^Tc-PYP-Negative Group N Group (*n* = 67)	^99m^Tc-PYP-Positive Group P Group (*n* = 9)	*p*-Value
Heart rate (bpm)	76	81	0.847
Atrial fibrillation/flutter (*n*, %)	6, 9.0	1, 11.1	0.836
Low voltage (*n*, %)	1, 1.5	1, 11.1	0.096
Left ventricular hypertrophy (*n*, %)	30, 44.8	1, 11.1	0.046
First-degree atrioventricular block (*n*, %)	13, 19.4	3, 33.3	<0.001
Left bundle branch block (*n*, %)	7, 10.4	2, 22.2	0.321
PQ duration (msec)	178	190	0.116
QRS duration (msec)	100	98	0.173
QTc duration (msec)	422	456	0.001

Continuous data are expressed as the median. *p*-values were determined using the Mann–Whitney U test.

**Table 4:** Echocardiographic examinations between the two groups.

	^99m^Tc-PYP-Negative Group N Group (*n* = 67)	^99m^Tc-PYP-Positive Group P Group (*n* = 9)	*p*-Value
Left atrial diameter (mm)	40.7	44.7	0.156
Left ventricular end-diastolic diameter (mm)	49.2	44.4	0.130
Left ventricular end-systolic diameter (mm)	33.3	31.6	0.631
Interventricular septal thickness at end diastole (mm)	13.7	16.1	0.084
Posterior wall thickness at end diastole (mm)	12.5	15.5	0.003
Left ventricular ejection fraction (%)	60.6	53.9	0.198
The percentage of HFpEF (*n*, %)	53, 79.1	6, 66.6	0.407

HFpEF: heart failure with preserved ejection fraction. Continuous data are expressed as the median. *p*-values were determined using the Mann–Whitney U test.

## Data Availability

The datasets used and/or analyzed during the current study are available from the corresponding author on reasonable request. The data are not publicly available due to privacy or ethical restrictions.

## References

[1] Maurer M.S., Schwartz J.H., Gundapaneni B., Elliott P.M., Merlini G., Waddington-Cruz M., Kristen A.V., Grogan M., Witteles R., Damy T. (2018). Tafamidis Treatment for Patients with Transthyretin Amyloid Cardiomyopathy. N. Engl. J. Med..

[2] Kitaoka H., Izumi C., Izumiya Y., Inomata T., Ueda M., Kubo T., Koyama J., Sano M., Sekijima Y., Tahara N. (2020). JCS 2020 Guideline on Diagnosis and Treatment of Cardiac Amyloidosis. Circ. J..

[3] Lane T., Fontana M., Martinez-Naharro A., Quarta C.C., Whelan C.J., Petrie A., Rowczenio D.M., Gilbertson J.A., Hutt D.F., Rezk T. (2019). Natural History, Qoality of Life, and Outcome in Cardiac Tramsthyretin Amyloidosis. Circulation.

[4] Naiki H., Yamaguchi A., Sekijima Y., Ueda M., Ohashi K., Hatakeyama K., Ikeda Y., Hoshii Y., Shintani-Domoto Y., Miyagawa-Hayashino A. (2023). Steep increase in the number of transthyretin-positive cardiac biopsy cases in Japan: Evidence obtained by the nation-wide pathology consultation for the typing diagnosis of amyloidosis. Amyloid.

[5] Elliott P., Drachman B.M., Gottlieb S.S., Hoffman J.E., Hummel S.L., Lenihan D.J., Ebede B., Gundapaneni B., Li B., Sultan M.B. (2022). Long-term Survival with Tafamidis in Patients with Transthyretin Amyloid Cardiomyopathy. Circ. Heart Fail..

[6] Elliott P., Gundapaneni B., Sultan M.B., Ines M., Garcia-Pavia P. (2023). Improves long-tern survival with tafamidis treatment in patients with transthyretin amyloid cardiomyopathy and severe heart failure symptoms. Eur. J. Heart Fail..

[7] Inomata T., Tahara N., Nakamura K., Endo J., Ueda M., Ishii T., Kitano Y., Koyama J. (2021). Diagnosis of wild-type transthyretin amyloid cardiomyopathy in Japan: Red-flag symptom clusters and diagnostic algorithm. ESC Heart Fail..

[8] Usuku H., Takashio S., Yamamoto E., Kinoshita Y., Nishi M., Oike F., Marume K., Hirakawa K., Tabata N., Oda S. (2020). Usefulness of relative apical longitudinal strain index to predict positive ^99m^Tc-labeled pyrophosphate scintigraphy findings in advanced-age patients with suspected transthyretin amyloid cardiomyopathy. Echocardiography.

[9] Sokolow M., Lyon T.P. (1949). The ventricular complex in left ventricular hypertrophy as obtained by unipolar precordial and limb leads. Am. Heart J..

[10] Roguin A. (2011). Henry Cuthbert Bazett (1885–1950)—The man behind the QT interval correction formula. Pacing Clin. Electrophysiol..

[11] Tsutsui H., Ide T., Ito H., Kihara Y., Kinugawa K., Kinugawa S., Makaya M., Murohara T., Node K., Saito Y. (2021). JCS/JHFS 2021 Guideline Focused Update on Diagnosis and Treatment of Acute and Chronic Heart Failure. Circ. J..

[12] González-López E., Gallego-Delgado M., Guzzo-Merello G., de Haro-Del Moral F.J., Cobo-Marcos M., Robles C., Bornstein B., Salas C., Lara-Pezzi E., Alonso-Pulpon L. (2015). Wild-type transthyretin amyloidosis as a cause of heart failure with preserved ejection fraction. Eur. Heart J..

[13] Schiller N.B., Shah P.M., Crawford M., DeMaria A., Devereux R., Feigenbaum H., Gutgesell H., Reichek N., Sahn D., Schnittger I. (1989). Recommendations for quantitation of the left ventricle by two-dimensional echocardiography. J. Am. Soc. Echocardiogr..

[14] Koizumi M., Watanabe H., Kaneko Y., Iino K., Ishida M., Kosaka T., Motohashi Y., Ito H. (2012). Impact of obesity on plasma B-type natriuretic peptide levels in Japanese community-based subjects. Heart Vessels.

[15] Srisawasdi P., Vanavanan S., Charoenpanichkit C., Kroll M.H. (2010). The effect of renal dysfunction on BNP, NT-proBNP, and their ratio. Am. J. Clin. Pathol..

[16] Shinzato K., Takahashi Y., Yamaguchi T., Otsubo T., Nakashima K., Yoshioka G., Yokoi K., Tsuruta K., Osako R., Shichida S. (2025). Atrial amyloidosis identified by biopsy in atrial fibrillation: Prevalence and clinical presentation. Eur. Heart J..

[17] Donnellan E., Wazni O.M., Hanna M., Elshazly M.B., Puri R., Saliba W., Kanj M., Vakamudi S., Patel D.R., Baranowski B. (2020). Atrial Fibrillation in Transthrretin Cardiac Amyloidosis: Predictors, Prevalence, and Efficacy of Rhythm Control Strategies. JACC Clin. Electrophysiol..

[18] Hosono K., Kiuchi S., Ikeda T. (2023). Cardiac Amyloidosis Patient with Cardiac Conduction Disturbances. J. Clin. Med. Res..

[19] Chen R., Bai K., Lu F., Zhao Y., Pan Y., Wang F., Zhang L. (2019). Electrocardiographic left ventricular hypertrophy and mortality in an oldest-old hypertensive Chinese population. Clin. Interv. Aging.

[20] Pewsner D., Jüni P., Egger M., Battaglia M., Sundström J., Bachmann L.M. (2007). Accuracy of electrocardiography in diagnosis of left ventricular hypertrophy in arterial hypertension: Systematic review. BMJ.

[21] Cuddy S.A., Datar Y., Ovsak G., Saith S., Murphy S.P., Bay C.P., Haddad M., Lilleness B., Muralidhar V., Pipilas A. (2022). Optimal Echocardiographic Parameters to Improve the Diagnostic Yield of Tc-99m-Bone Avid Tracer Cardiac Scintigraphy for Transthyretin Cardiac Amyloidosis. Circ. Cardiovasc. Imaging.

[22] Oike F., Usuku H., Yamamoto E., Marume K., Takashio S., Ishii M., Tabata N., Fujisue K., Yamanaga K., Sueta D. (2022). Utility of left atrial and ventricular strain for diagnosis of transthyretin amyloid cardiomyopathy in aortic stenosis. ESC Heart Fail..

[23] Carroll J.D., Gaasch W.H., McAdam K.P. (1982). Amyloid cardiomyopathy: Characterization by a distinctive voltage/mass relation. Am. J. Cardiol..

[24] Sharma S., Labib S.B., Shah S.P. (2021). Electrocardiogram Criteria to Diagnose Cardiac Amyloidosis in Men with a Bundle Branch Block. Am. J. Cardiol..

[25] Pagura L., Porcari A., Cameli M., Biagini E., Canepa M., Crotti L., Imazio M., Forleo C., Pavasini R., Limongelli G. (2024). ECG/echo indexes in the diagnostic approach to amyloid cardiomyopathy: A head-to-head comparison from the AC-TIVE study. Eur. J. Intern. Med..

[26] Addison D., Slivnick J.A., Campbell C.M., Vallakati A., Jneid H., Schelbert E. (2021). Recent Advances and Current Dilemmas in the Diagnosis and Management of Transthyretin Cardiac Amyloidosis. J. Am. Heart Assoc..

[27] Bukhari S., Khan B. (2023). Prevalence of ventricular arrhythmias and role of implantable cardioverter-defibrillator in cardiac amyloidosis. J. Cardiol..

[28] Brown M.T., Yalamanchili S., Evans S.T., Ram P., Blank E.A., Lyle M.A., Merchant F.M., Bhatt K.N. (2022). Ventricular arrhythmia burden and implantable cardioverter-defibrillator outcomes in transthyretin cardiac amyloidosis. Pacing Clin. Electrophysiol..

[29] Del Duca F., Ghamlouch A., Manetti A.C., Napoletano G., Sonnini E., Treves B., De Matteis A., La Russa R., Sheppard M.N., Fineschi V. (2024). Sudden Cardiac Death, Post-Mortem Investigation: A Proposing Panel of First Line and Second Line Genetic Tests. J. Pers. Med..

[30] Hafeez A.S., Bavry A.A. (2020). Diagnosis of Transthyretin Amyloid Cardiomyopathy. Cardiol. Ther..

[31] Yamasaki H., Kondo H., Shiroo T., Iwata N., Masuda T., Makita T., Iwabuchi Y., Tanazawa K., Takahashi M., Ono Y. (2024). Efficacy of Computed Tomography-Based Evaluation of Myocardial Extracellular Volume Combined with Red Flags for Early Screening of Concealed Cardiac Amyloidosis in Patients with Atrial Fibrillation. Circ. J..

